# Cardiac diffusion-weighted and tensor imaging: A consensus statement from the special interest group of the Society for Cardiovascular Magnetic Resonance

**DOI:** 10.1016/j.jocmr.2024.101109

**Published:** 2024-10-22

**Authors:** Erica Dall’Armellina, Daniel B. Ennis, Leon Axel, Pierre Croisille, Pedro F. Ferreira, Alexander Gotschy, David Lohr, Kevin Moulin, Christopher T. Nguyen, Sonja Nielles-Vallespin, William Romero, Andrew D. Scott, Christian Stoeck, Irvin Teh, Elizabeth M. Tunnicliffe, Magalie Viallon, Victoria Wang, Alistair A. Young, Jürgen E. Schneider, David E. Sosnovik

**Affiliations:** a Biomedical Imaging Science Department, Leeds Institute of Cardiovascular and Metabolic Medicine, Leeds, UK; b Department of Radiology, Stanford University, Stanford, California, USA; c Department of Radiology, NYU Grossman School of Medicine, New York, New York, USA; d Royal Brompton Hospital and National Heart and Lung Institute, Imperial College London, London, UK; e Chair of Molecular and Cellular Imaging, Comprehensive Heart Failure Center Wuerzburg (CHFC), University Hospital Wuerzburg, Wuerzburg, Germany; f Department of Cardiology, Boston Children's Hospital, Harvard Medical School, Boston, Massachusetts, USA; g Harvard Medical School, Boston, Massachusetts, USA; h Oxford Centre for Clinical Magnetic Resonance Research, Division of Cardiovascular Medicine, Radcliffe Department of Medicine, University of Oxford and Oxford NIHR Biomedical Research Centre, University of Oxford, Oxford, UK; i Kings College, London, UK; j Martinos Center for Biomedical Imaging and Cardiovascular Research Center, Massachusetts General Hospital, Harvard Medical School, Boston, Massachusetts, USA; k Department of Cardiology, University Hospital Zurich, Zurich, Switzerland; l Division of Cardiology, Department of Internal Medicine, NYU Grossman School of Medicine, New York, New York, USA; m Center for Preclinical Development, University of Zurich and University Hospital Zurich, Zurich, Switzerland; n Institute for Biomedical Engineering, University and ETH Zurich, Zurich, Switzerland; o Department of Biomedical Engineering, Case Western Reserve University and Lerner Research Institute Cleveland Clinic, Cleveland, Ohio, USA; p Cardiovascular Innovation Research Center, Heart, Vascular, and Thoracic Institute, Cleveland Clinic, Cleveland, Ohio, USA; q University of Lyon, UJM-Saint-Etienne, INSA, CNRS UMR 5520, INSERM U1206, CREATIS, F-42270, Saint-Etienne, France; r Department of Radiology, University Hospital Saint-Etienne, F-42055 Saint-Etienne, France

**Keywords:** Cardiovascular magnetic resonance, Cardiac diffusion imaging, Cardiac diffusion tensor imaging, Myocardial structure, Recommendations, Consensus

## Abstract

Thanks to recent developments in cardiovascular magnetic resonance (CMR), cardiac diffusion-weighted magnetic resonance is fast emerging in a range of clinical applications. Cardiac diffusion-weighted imaging (cDWI) and diffusion tensor imaging (cDTI) now enable investigators and clinicians to assess and quantify the tridimensional microstructure of the heart. Free-contrast DWI is uniquely sensitized to the presence and displacement of water molecules within the myocardial tissue, including the intracellular, extracellular, and intravascular spaces. CMR can determine changes in microstructure by quantifying: a) mean diffusivity (MD)—measuring the magnitude of diffusion; b) fractional anisotropy (FA)—specifying the directionality of diffusion; c) helix angle (HA) and transverse angle (TA)—indicating the orientation of the cardiomyocytes; d) absolute sheetlet angle (E2A) and E2A mobility—measuring the alignment and systolic-diastolic mobility of the sheetlets, respectively.

This document provides recommendations for both clinical and research cDWI and cDTI, based on published evidence when available and expert consensus when not. It introduces the cardiac microstructure focusing on the cardiomyocytes and their role in cardiac physiology and pathophysiology. It highlights methods, observations, and recommendations in terminology, acquisition schemes, postprocessing pipelines, data analysis, and interpretation of the different biomarkers. Despite the ongoing challenges discussed in the document and the need for ongoing technical improvements, it is clear that cDTI is indeed feasible, can be accurately and reproducibly performed and, most importantly, can provide unique insights into myocardial pathophysiology.

## 1 Introduction

The Society of Cardiovascular Magnetic Resonance (SCMR) Cardiac Diffusion Special Interest Group wrote this SCMR-endorsed consensus paper to provide recommendations for the research and clinical application of cardiac diffusion-weighted imaging (cDWI) and diffusion tensor imaging (cDTI). The special interest group seeks to promote, validate, and standardize cDWI/cDTI, and anticipates that this document will be of value to the scientific community, equipment manufacturers, software developers, and clinicians. The document presented here cites published evidence when available and provides expert consensus where incomplete. This consensus document is not intended to provide a comprehensive review of the field, and the interested reader is referred to several recently published reviews [1], [2], [3].

Valuable research has been performed with diffusion-weighted magnetic resonance (MR) imaging of the heart for three decades, but until recently its complexity has limited its potential for routine use. However, recent advances in MR hardware, acquisition strategies, and postprocessing have now demonstrated the potential of cDWI/cDTI in a range of clinical applications. The aim of this document is two-fold: i) to introduce the technique to those with little or no experience in diffusion cardiovascular magnetic resonance (CMR); and ii) to establish a common framework within the CMR community to move the technique forward.

The consensus begins with an executive summary of the recommendations. This is followed by the main document, where a more detailed exploration of the field is proposed and the recommendations discussed. The main consensus statement begins with a brief description of the cellular microstructure of the heart and its role in cardiac physiology and pathophysiology. A set of recommended terms, uniquely suited to the microstructure of the heart and correlated with well-defined histological features, is then provided. The rapid motion and deformation of the heart makes the execution of cDWI challenging: acquisition schemes that overcome this are reviewed, and recommendations for their use are provided. The postprocessing and analysis of diffusion-weighted images is a very rich and dynamic area, and here we make recommendations for a common set of basic analyses and metrics to report. The relationship of these parameters to cardiac pathophysiology and their interpretation in the context of existing CMR parameters is then discussed. The large body of research validating the accuracy of diffusion CMR and justifying the recommendations made is then reviewed. The document ends with a review of the ongoing challenges in diffusion CMR and a set of recommendations for the research and clinical communities, and for industry.

## 2 Executive summary of recommendations

The summary below provides a synopsis of selected recommendations made in the main document. This summary provides a basic scaffold for the reader, and it is not intended to be comprehensive.

### 2.1 Biology of cardiac microstructure

• When describing myocardial microstructure, in the context of cardiac DTI, the terms “fiber” and “myofiber” should be avoided. While “fiber-like” structures may be seen at the histological scale and single-cell level, distinct individual physical fibers cannot be inferred from DTI-derived metrics, due to the continuously branching pattern of the cardiomyocytes in the heart. In this regard, the myocardium differs significantly from skeletal muscle, where DTI-derived tracts do correlate with distinct anatomical fibers.
• The microstructure of the heart should be taken into account when studying cardiac mechanics, including abnormalities of contraction, relaxation, and dyssynchrony.
• Strategies to regenerate lost myocardium should account for the anisotropic nature of the myocardium and use techniques, such as cDTI, to assess the orientation and organization of newly formed cardiomyocytes.
• Personalized computational models of electrical conduction and arrhythmogenesis in the heart should incorporate the impact of cardiomyocyte orientation.

### 2.2 Recommended terminology for diffusion imaging of the heart

**Fig. 1:**
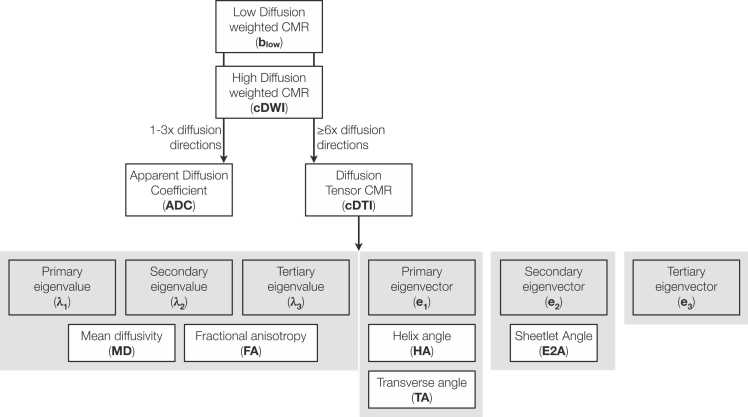
**Diffusion (cDWI) CMR terminology.** Low and high diffusion-weighted images are acquired, then used to reconstruct ADC or cDTI maps. Primary, secondary, and tertiary eigenvalues and eigenvectors are calculated from the diffusion tensor and used to derive commonly used metrics of diffusivity and microstructure in the heart. *CMR* cardiovascular magnetic resonance, *cDTI* cardiac diffusion tensor imaging, *cDWI* cardiac diffusion-weighted imaging

• The following terms are recommended, based on prior and current use: cardiac diffusion weighted imaging (cDWI) and cardiac diffusion tensor imaging (cDTI)
• Other favored terms to describe diffusion or diffusion tensor myocardial microstructure include: mean diffusivity (MD), fractional anisotropy (FA), helix angle (HA), and absolute sheetlet angle (E2A) (refer to Fig. 1, Table 1, Table 2)

**Table 1:** Terminology of commonly reported cDTI parameters.

Preferred terms	Units	Expected values	Definition*
Diffusion tensor (D)	×10^−3^ mm^2^s^−1^ (µm ms^−1^)	3 × 3 matrix	A rank of 2, symmetric and positive semi-definite tensor that can be expressed as a 3 × 3 matrix.
Primary eigenvalue (λ_1_)	×10^−3^ mm^2^s^−1^ (µm ms^−1^)	<3 × 10^−3^ mm^2^s^−1^	Apparent diffusivity in the direction of the primary eigenvector e_1._ Aligns with the mean intravoxel cardiomyocyte long axis.
Primary eigenvector (e_1_)	Unitless		Orientation of the diffusion along λ_1_. Aligns with the mean intravoxel cardiomyocyte long axis.
Secondary eigenvalue (λ_2_)	×10^−3^ mm^2^s^−1^ (µm ms^−1^)	<λ_1_	Apparent diffusivity in mean intravoxel sheetlet orientation perpendicular to the cardiomyocyte orientation within a sheetlet.
Secondary eigenvector (e_2_)	Unitless		Orientation of the diffusion along λ_2_. Lies perpendicular to the mean cardiomyocyte orientation within the mean intravoxel sheetlet plane.
Third eigenvalue (λ_3_)	×10^−3^ mm^2^s^−1^ (µm ms^−1^)	<λ_2_	Apparent diffusivity in the direction perpendicular to the mean intravoxel sheetlet plane.
Third eigenvector (e_3_)	Unitless		Orientation of the diffusion along λ_3_. Defines the sheetlet normal vector.
Apparent diffusion coefficient (ADC)	×10^−3^ mm^2^s^−1^ (µm ms^−1^)	<3 × 10^−3^ mm^2^s^−1^	The measured diffusion coefficient in a given orientation.
Mean ADC†	×10^−3^ mm^2^s^−1^ (µm ms^−1^)	<3 × 10^−3^ mm^2^s^−1^ (usually ∼1 × 10^−3^ mm^2^s^−1^)	The mean ADC value calculated from cDWI data acquired with diffusion encoding in three perpendicular directions.
Mean diffusivity (MD)†	×10^−3^ mm^2^s^−1^ (µm ms^−1^)	<3 × 10^−3^ mm^2^s^−1^ (usually ∼1 × 10^−3^ mm^2^s^−1^)	Average of the eigenvalues obtained from cDTI. A measure of mean diffusion magnitude.
Fractional anisotropy (FA)	Unitless	0–1 (usually 0.3–0.6)	Measure of anisotropy, i.e., directionality of diffusion (FA = 0, uniform diffusion in all directions).
Tensor mode (mode)	Unitless	−1 to +1	Measure of the kind of anisotropy. Ranges from a stick (mode = 1) to a disk (mode = −1)
Helix Angle (HA)	˚ (degrees)	Typically ∼ −60˚ to +60˚ Bounded by:−90˚ to +90˚	The angle between the projection of the first eigenvector into the local epicardial plane and the local short axis (imaging) plane.
Transverse angle	˚ (degrees)	∼0˚	The angle between the projection of the first eigenvector into the local radial-circumferential plane and the circumferential vector.
E2A	˚ (degrees)	E2A (systole) > E2A (diastole)	Calculated from the second eigenvector. Absolute values are typically quoted.

* Any reported measure of diffusion >3 × 10^−3^ mm^2^s^−1^ exceeds that of free water at 37°C and suggests corruption by noise, motion artifacts, or other errors

† ADC is the apparent diffusion coefficient of a single diffusion-weighted direction, while MD is the averaged ADC in three orthogonal planes (it is also the average of the eigenvalues, which are also orthogonal, from the diffusion tensor). In general, MD is seen to be more robust and should be preferentially reported *cDWI* cardiac diffusion-weighted imaging, *cDTI* cardiac diffusion tensor imaging

**Table 2:** Terminology of acquisition parameters.

Parameter	Units	Typical values (in vivo)	Definition	Notes
b	s mm^−2^ (ms µm^−2^ = 10^−3^ × s mm^−2^)	250–750 s/mm^−2^	Factor that reflects the strength and timing of the gradients used to generate diffusion-weighted images [4]	Magnitude of diffusion-weighted contrast
δ	ms	∼1–10 s ms	Duration of the diffusion encoding gradient	
Δ	ms	STEAM—1 cardiac cycle ∼1000 ms M1M2-Comp SE—not well defined, but in the order of 10s of ms	Time between paired diffusion-encoding gradients.	Not to be confused with TM for STEAM.
M0, M1,M2		M0 = M1 = M2 = 0	Order of motion compensation in diffusion gradients.	Used for spin echo sequences
G	mT/m	40–80	Amplitude of diffusion-encoding gradients.	Often times, the maximum G is reported.
TM	ms	∼Δ	Time magnetization is stored in the longitudinal direction between the second and third RF pulses.	STEAM only, increasing TM results in more T1 weighting.
TE	ms	∼25 ms STEAM ∼75 ms M1M2-SE	Echo time	Shorter TEs improve SNR
TR	ms	STEAM - Two cardiac cycles or ∼2 s M1M2-Comp SE—1–4 cardiac cycles or ∼1–4 s	Time between first 90˚ RF pulses acting on the same slice in successive sequence repeats, Fig. 2	STEAM requires two cardiac cycles; M1M2-Comp SE uses one if breath held, but ∼4 if free-breathing
Trigger time (TT)	ms	0 ≤TR < 1 RR interval	Time from the R-wave to the time of the acquisition of the central k-space data.	

*STEAM* Stimulated echo acquisition mode, *TM* mixing time, *TE* echo time, *TR* repetition time, *SE* spin echo, *RF* radio frequency
• To describe the tridimensional (3D) pattern of cardiomyocytes as assessed by tractography, terms such as aggregates, streamlines, or tracts, could be used instead of fiber.

### 2.3 Image acquisition—pulse sequences

• Only cDWI strategies designed to specifically compensate for cardiac motion during diffusion encoding should be used.
• All acquisitions should involve prospective cardiac triggering.
• Stimulated echo acquisition mode (STEAM) data have to be acquired during breath-holds. Spin echo (SE) acquisitions can be acquired during breath-holds or free-breathing, with either prospective or retrospective respiratory triggering.

### 2.4 Image acquisition—sequence parameters for cDTI

• Cardiac DTI requires a minimum of six non-collinear diffusion-encoding directions and one reference image. In practice, it is considered safest to acquire more than six directions for acquisition robustness (in case one or more directions are corrupted).
• The b-value of the diffusion-encoded images should be 350-500 s/mm^2^. A b-value = 0 is not recommended in the reference image, with a minimum b-value around 50 s/mm^2^ to avoid confounders due to perfusion (Table 2).
• Single-shot echoplanar imaging (EPI) readouts with a maximal slice thickness of 8 mm in the short-axis plane, and an in-plane resolution between 2.5 × 2.5 mm^2^ and 3.0 × 3.0 mm^2^ are recommended with higher than 2.5 mm^2^ encouraged, unless constrained by signal-to-noise ratio (SNR).
• SE sequences should employ diffusion-encoding gradients designed to null the first and second moments of motion and should be acquired at mid systole (motion-compensated spin echo [MCSE]).
• STEAM sequences can be acquired at any phase of the cardiac cycle. However, the long mixing time (TM) in a stimulated echo sequence exposes the diffusing protons to myocardial deformation/strain. The impact of this on the diffusion signal is negligible at certain “sweet spots” of the cardiac cycle, and likely small at other phases of the cardiac cycle, but remains incompletely characterized. This uncertainty and knowledge gap should be addressed with appropriate simulation and phantom studies and be acknowledged in relevant publications.

### 2.5 Image acquisition—hardware considerations

• Images may be acquired at 1.5T or 3T, although a preference is emerging for 3T at most centers, owing mostly to improved SNR.
• If the maximal gradient system on the scanner is 80 mT/m or higher, either a SE or STEAM sequence can be used.
• If the maximal gradient strength on the system is < 80mT/m, the echo time (TE) on an SE sequence is longer; in this context, STEAM sequences are generally preferred.
• The number of slices acquired, their location, and the gap between them should be tailored to the pathology being studied and the question being asked. In general, confidence in a study’s conclusion will be increased if more than one slice is acquired.

### 2.6 Image quality and optimization

• To establish sequence-specific normal values and optimize data acquisition, cDWI/cDTI data should be acquired in 20 normal volunteers.
• The researcher and clinical practitioner using cDWI/cDTI should be able to recognize artifacts in the raw images and attempt to remedy them.
• Those using cDTI should be familiar with the range of normal values for MD, FA, HA, and E2A, and compare values acquired on their scanners against the reference values provided in this document (Table 4).
• If breath-hold capacity is limited, then a free-breathing SE approach should be strongly considered. Likewise, in case of cardiac arrhythmia, a SE approach is preferred.

### 2.7 Postprocessing and reporting

• Cardiac trabeculations should not be included in the analysis of MD, FA, HA, or E2A.
• Dedicated software should be used to analyze cDWI/cDTI data.
• Corrupted raw images should be manually or automatically identified and removed before further processing. Spatial co-registration of the images should be performed prior to further analysis.
• Data can be analyzed in each segment of the myocardium individually or in a single region of interest (ROI) placed in the interventricular septum, as dictated by the condition being studied and questions being asked. The septal ROI should ideally start/end at the RV insertion points and include the full thickness of the myocardium (endocardium to epicardium) to derive HA and E2A. Very small ROIs should be avoided to minimize sampling errors [5]. The technique used for analysis should be clearly stated and justified.
• To provide HA and E2A, cardiomyocyte (e1) and sheetlet angles (e2) should be calculated and then displayed relative to a cardiac coordinate system and not standard Cartesian axes.
• No particular color scale is preferred. However, color scale bars should accompany all images and be clearly labeled. A cyclic color scale should be used for HA maps and other cyclical angular maps, to avoid false discontinuities.

### 2.8 Scientific and clinical interpretation

• The normal values of MD and FA depend on the sequence used—stimulated or SE. The cDTI user should be familiar with the biophysical basis for this and the normal values for the sequence they use (Table 3).

**Table 3:** Typical acquisition protocols.

	TR/ms	BW/pixel/Hz	Typical b low values/s/mm^2^	Typical b-values/s/mm^2^	Fat saturation	Respiratory compensation		FOV/mm (matrix size)	Typical TE/ms
STEAM	2RR intervals*	2480	50–100	450–500	Yes	Breath-hold	Full FOV†	360 × 170 (144 × 108)	44‡
MCSE	1 RR interval or more if slice interleaving	2480	50–100	450–500	Yes	Breath-hold or free-breathing	Full FOV†	As above	70–80§

*TR* trigger time, *FOV* field of view, *TE* echo time, *STEAM* stimulated echo acquisition mode, *MCSE* motion-compensated spin echo, *BW* Bandwidth

* As STEAM is acquired during breath-holding, slice interleaving is not typically available

**Fig. 2:**
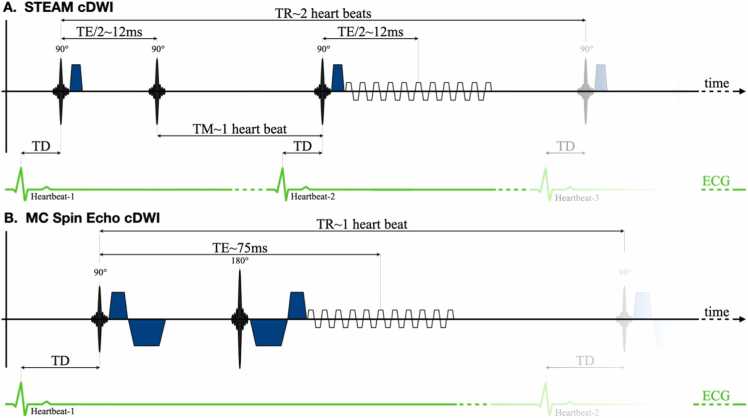
**Cardiac diffusion-weighted sequences.** The two most common cardiac diffusion-weighted imaging (cDWI) pulse sequences both use ECG triggering, diffusion-weighting gradients (dark blue), and single-shot echoplanar imaging (EPI) readouts. (A) Stimulated echo acquisition mode (STEAM) cDWI uses smaller diffusion-encoding gradients and longer diffusion mixing times (TM), which requires two heartbeats per image. (B) Motion-compensated (MC) spin echo (SE) uses larger diffusion-encoding gradients applied within a single heartbeat. *ECG* electrocardiogram, *TR* repetition time, *TE* echo time, *TD* trigger delay

† Reduced FOV may provide an option for smaller matrix size and a shorter TE of 24–30 ms (Fig. 2)

‡ TE is primarily related to the readout for STEAM, as the diffusion-encoding gradients are very short for clinically relevant b-values

§ Requires 80 mT/m gradients; lower-strength gradient systems lead to unacceptably long echo times
• Changes in diffusion-derived metrics are seen in many conditions, but are not specific for a particular etiology. Acute and chronic injury are both often characterized by an increase in MD, reduction in FA, perturbations in HA (reductions in range, coherence and transmural slope), and reductions in E2A (sheetlet) mobility.
• Changes in MD, FA, HA, and E2A are not specific and should be interpreted in the context of other common CMR-derived parameters, such as T1, T2, extracellular volume (ECV), and strain.
• The correlation of DTI-derived metrics with histology has been performed in healthy animal hearts and in animal models of myocardial infarction (MI) but not in other conditions, such as hypertrophic cardiomyopathy (HCM). The use of DTI-derived parameters, such as MD and FA, to describe histological patterns should be used with caution unless direct histological correlation has been performed. Until direct histological comparisons can be performed, the use of terminology such as “disarray” based solely on changes of FA/MD in HCM is discouraged.
• 3D tractography provides a valuable tool to comprehensively visualize long-range cardiomyocyte orientation and connectivity across the entire heart. Several quantitative metrics can be derived from these tractograms, but remain experimental. Standard analysis of cardiomyocyte orientation should be performed at present using 2D HA maps.

#### 2.9 Rigor and reproducibility

• Familiarity with the extremely large body of *ex vivo,* preclinical, and phantom-based studies that have confirmed the accuracy of cDTI to characterize myocardial microstructure is needed to form a basis for interpreting in vivo results.
• Preclinical and phantom-based studies are strongly encouraged and remain extremely valuable for the development and validation of new sequences/techniques and to correlate clinical observations with microscopic (cellular level) changes.
• Clinical studies to confirm single-site observations should be encouraged across the full range of cardiac conditions.
• Multi-center studies are strongly encouraged, particularly in conditions where cDTI has shown the potential to change patient management.

#### 2.10 Challenges and opportunities

• cDTI remains a very rich area for research and development. Current areas of particular need include denoising, extending anatomical coverage, increasing spatial resolution, and reducing scan time.
• Artifacts and distortion remain major problems in cDTI, and a substantial effort should be made to address this.
• The use of artificial intelligence (AI)-based approaches to process the diffusion-weighted data, reject corrupted data, reduce noise, and accelerate analysis is very promising, but requires extensive validation.
• At the present time, no single technique has been shown to reproducibly improve the accuracy of cDTI data, while simultaneously reducing scan time and/or extending coverage. The Special Interest Group encourages a major research endeavor in this area.

#### 2.11 Recommendations for the scanner manufacturers and software vendors

• The accuracy and value of cDTI has now been definitively demonstrated in a range of clinical applications. We call upon the scanner manufacturers to provide the broad clinical and research CMR community with basic versions of diffusion-encoded stimulated and SE sequences.
• We further call on the scanner manufacturers to provide tools for the inline calculation of MD, FA, HA, and E2A in the heart.
• We encourage the vendors of CMR analysis platform to develop modules to process cDTI data, including MD, FA, HA, E2A, and other advanced metrics of diffusion in the heart.

A further exploration of these recommendations, their scientific basis, and justification is provided in the main document below.

## 3 Main document and recommendations

### 3.1 Biological relevance of cardiac microstructure

#### 3.1.1 Cells of the heart

Approximately two billion cardiomyocytes compose the adult human heart and are organized into a remarkable mechanical and electrical syncytium that gives rise to highly coordinated contraction and the efficient ejection of blood [3]. The myocardium is composed of many kinds of cells, including cardiomyocytes, fibroblasts, endothelial cells, pericytes, smooth muscle cells, immune cells, adipocytes, mesothelial cells, and neuronal cells [6]. Due to the large size of the cardiomyocytes (∼100 µm long and ∼20 µm diameter) [7], they account for most of the heart’s cellular volume, but the majority of cells in the heart are non-cardiomyocytes. Human cardiomyocytes are diploid, reflecting the need to maintain a uniquely high mitochondrial content and the complex sarcomeric apparatus needed for contraction. Cardiomyocytes form a continuously branching syncytium, surrounded by a complex of lymph vessels, nerves, and blood vessels, all bound together with a fibrous (primarily collagen) extracellular matrix. The complex, but highly organized, network of cardiomyocytes forms an excitable bulk material that is capable of the efficient ejection of blood.

#### 3.1.2 Myocardial microstructure

The arrangement of cardiomyocytes throughout the heart is complex, with several levels of organization. Cardiomyocytes form a continuously branching syncytium and are joined together end-to-end in a “split and merge” manner, so that they do not form distinct continuous “fibers” as seen in skeletal muscle, nerves, or connective tissue. The local orientation of the cardiomyocytes produces a visible grain (or pattern) that changes orientation systematically from epicardium to endocardium and remains largely in a plane parallel to the heart surfaces. Streeter et al. first defined a coordinate system for measuring the orientation of the grain relative to the heart’s geometry and measured an obliquely oriented (in opposite directions) grain in the sub-epicardium and sub-endocardium, a circumferential grain in the mid-wall, and a smooth transition between [8]. This transmural pattern is conserved across mammalian species ranging from mice to humans [9], [10], [11], [12], [13], [14], [15], [16].

In addition to their end-to-end junctions, the cardiomyocytes are composited to form sheetlet structures by a hierarchy of perimysial connective tissue fibers [17] constituting the extracellular matrix of the heart wall. Cardiomyocytes form sheetlet layers 4–6 cells thick [17] that are essential to ventricular wall thickening and also underlie anisotropic electrical conduction [18]. The “spaces” between the sheetlets accommodate cell-to-cell sliding necessary for ventricular wall thickening [3].

The regenerative capacity of the human heart is extremely limited and attempts to regenerate lost myocardium through endogenous repair mechanisms, exogenous cells, or tissue patches will need to recapitulate the embryonic development of its microstructure [9], [16]. The structure of the myocardium changes during embryologic development, with a transition from an initially “spongy” (more isotropic) state early in development to a more “compacted” (more anisotropic) state later. The typical anisotropic architecture of the heart and the characteristic oblique orientation of the subendocardial and subepicardial cardiomyocytes develop in the second trimester [19].

#### 3.1.3 Mechanics, conduction, and microstructure

Individual cardiomyocytes undergo relatively little shortening (∼12%–14%) at peak contraction [20], whereas the left ventricular wall thickening (radial) strain can be quite high (>25%). These comparatively small cardiomyocyte strains are amplified to produce large changes in myocardial strain and bulk contraction, owing to the complex 3D architecture of the cardiomyocytes comprising the myocardium [3]. While there are no radially oriented cardiomyocytes in the heart, their complex microstructural arrangement produces significant strain in the radial direction.

The systematic change in cardiomyocyte orientation across the LV heart wall allows greater overall wall contraction and more uniform energy utilization [21]. The obliquely oriented cardiomyocytes near the epicardial and endocardial surfaces of the LV contribute to longitudinal contraction and the more circumferentially oriented mid-wall cardiomyocytes largely produce circumferential contraction. The greater effective lever arm of the obliquely oriented subepicardial cardiomyocytes leads to a net torsional component of cardiac contraction. It is observed that the base of the LV rotates clockwise relative to the counter-clockwise rotating apex (when viewed from apex to base). There are several measures of rotational mechanics, including twist, torsion, and shear [22].

The sheetlet structure of the heart wall, where the potential spaces between sheetlets permit greater shear along the direction perpendicular to the sheetlet planes, critically underlies transmural thickening [23]. There are effectively two “interpenetrating” families of sheetlets, which are oriented similarly near the surfaces of the heart wall but are separated from each other near the mid-wall [17], [24]. The presence of these two families of sheetlets may make the conventional use of only six diffusion-sensitization directions inadequate to capture them. The dynamic rotation of these sheetlets across the cardiac cycle plays an important role in the contraction and relaxation of the myocardium [3].

The myocardium (unlike skeletal muscle) has an elaborate system of gap junctions and connexins, which allows cell-to-cell conduction to produce coordinated contraction. This electrical conduction is fastest along the direction of the cardiomyocytes, slower within the sheets, and slowest between sheets, in an approximate 4:2:1 ratio [18]. Mathematical models and preclinical studies of electrical activation in the heart have shown that incorporating myocardial anisotropy into the model significantly affects electrical conduction in the heart. Models to predict mechanics and arrhythmogenesis in the left ventricle are increasingly incorporating microstructural information, such as cardiomyocyte orientation, as a variable [25], [26], [27], further demonstrating the value of the approach.

### 3.2 Recommended terminology

Consistent terminology is important to avoid confusion and allow the field to develop. While some fields are limited by the terminology used by manufacturers, cardiac diffusion imaging is still relatively unencumbered. (Refer to Fig. 1, Table 1, Table 2)

*Diffusion* is the thermally driven Brownian motion of molecules, and in this context refers to the self-diffusion of water molecules in tissue.

*Diffusion contrast* is possible when diffusion-encoding gradients are applied and underlies both diffusion-weighted imaging (DWI) and diffusion tensor imaging (DTI).

*Diffusion-weighted imaging (DWI)*—MR imaging made sensitive to diffusion of water molecules in tissue, owing to the application of diffusion-encoding gradients. Termed “cardiac diffusion-weighted imaging” (cDWI) when used in the heart and preferred over terms such as D-CMR (diffusion cardiovascular magnetic resonance) or DW-CMR (diffusion-weighted CMR).

*Diffusion tensor imaging*—A cDWI method where diffusion weighting is applied in at least six non-collinear directions plus a reference scan, sufficient to determine the diffusion tensor. The diffusion tensor allows the preferred directions of water diffusion (termed eigenvectors) and the magnitude of diffusion in these directions (termed eigenvalues) to be determined and is of major utility in anisotropic tissues.

### 3.3 Acquisition techniques

#### 3.3.1 Diffusion-encoding pulse sequences

The most basic diffusion-encoding waveform, the monopolar experiment is composed of two identical-strength strong gradient lobes [28]. The first gradient lobe encodes the spins with a spatially dependent phase. Owing to either a refocusing pulse or a change in the gradient polarity, the second gradient lobe effectively decodes this spatially dependent phase. Any net spin displacement in the interval between the two gradients leads to incomplete rephasing. The displacement of spins due to the diffusion process leads to an intravoxel phase distribution that has a residual phase. At a voxel scale, this intravoxel phase distribution generates a signal attenuation in a similar fashion to the T2 effect. The incremental attenuation in the signal is given by:

(1) *S*=*S*_0_*e*^−*bD*^

*S* is the image pixel (voxel) intensity, which depends on the inherent signal intensity (*S*_0_), *D* is the intravoxel diffusion coefficient (mm^2^/s or µm^2^/ms), and *b* is the so-called b-value (s/mm^2^), which mathematically summarizes the timing and amplitude of the applied diffusion gradients. The b-value, which describes the strength of diffusion encoding, is dependent on the square of the applied gradient, and strong gradients can, therefore, facilitate more advanced schemes of diffusion encoding. The diffusion gradient waveforms used to achieve a specific b-value depend upon the choice of the SE or stimulated echo approach.

If the voxel undergoes bulk translation, all intravoxel spins are moved by the same amount, leading to a net intravoxel phase shift, but this has no effect on the signal magnitude. However, if the voxel undergoes deformation, then the intravoxel spins may be moved by different amounts, leading to an additional intravoxel phase distribution and leading to an unwanted signal attenuation [29], [30]. For this reason, specialized diffusion-encoding methods are needed when estimating diffusion in the deforming heart.

The key challenge in cardiac DWI lies in detecting spin displacements on the micrometer scale during bulk motion of the heart on the scale of many millimeters. Diffusing water moves randomly, with a final displacement influenced by the diffusion coefficient of water among nearby cellular structures and boundaries. In contrast, cardiac contraction is periodic, with each point in the heart following a smooth, consistent trajectory during contraction under sinus rhythm and constant heart rate, defined by velocities and accelerations. These different and separable motion characteristics have paved the way for spin echo and stimulated echo approaches that enable cardiac DWI.

##### 3.3.1.1 Stimulated echo acquisition mode (STEAM)

STEAM DWI/DTI is the earliest [31], [32] method used for cardiac diffusion imaging in vivo. The STEAM technique splits the diffusion encoding between two adjacent cardiac cycles (Fig. 2). The diffusion gradients are each timed to occur at an identical trigger delay (TD) in adjacent cardiac cycles, mitigating signal loss due to cardiac motion during the diffusion encoding and avoiding the need for motion compensation. After the first diffusion-encoding gradient, the magnetization is “stored” along the z-axis with a radio frequency (RF) pulse. Thereafter, the magnetization decays according to T1 during the TM, approximately equivalent to the RR interval. The effective diffusion sensitivity, or b-value, of this monopolar diffusion experiment is given by:

(2) $$b=\gamma^{2}\delta^{2}G^{2}\left(∆-\frac{\delta }{3}\right)$$

*STEAM advantages* – The water molecules diffuse for a relatively long time (∆, one RR interval), which has the advantage of providing comparatively strong diffusion-weighted contrast while only needing relatively short, lower-strength encoding gradients. The diffusion-encoding gradient duration (δ) may be ∼2–5 ms, with near-maximum gradient amplitudes (G) of 40–80 mT/m. These short diffusion-encoding gradients also result in short TEs (TE <25 ms) [33], thereby avoiding severe T2-related signal decay (which is much faster in the heart compared to, for example, the brain). The long ∆ used also means that the length scale of the microscopic structures described by the STEAM diffusion tensor is larger than those from SE methods [34].

*STEAM limitations* – Relative to SE, STEAM results in a 50% loss of SNR, and the SNR then decays with T1 between the second and third RF pulses. Splitting diffusion preparation over two cardiac cycles also further reduces the SNR efficiency relative to SE. Consequently, STEAM offers diffusion-encoding robustness at the expense of lower intrinsic SNR efficiency.

Other limitations of the STEAM method include the dependence of the b-value on the heart rate (i.e., heart rate changes cause changes in ∆). In addition, the minimum b-value of a STEAM sequence is substantial, owing to spoiler gradients used to spoil the free induction decay signal from the third RF pulse, meaning that true “b0” images are unavailable [33], [35]. Furthermore, the assumption that the heart is in the same state at the same TD in two successive cardiac cycles makes the STEAM sequence sensitive to arrhythmia, and also means that studies require breath-holding or guided breathing.

A further limitation of the STEAM sequence is the effect of tissue strain (deformation) during the diffusion time, ∆, on the measured diffusion [36]. The magnitude of this effect is thought to be greater when encoding at peak systole than in diastasis, and some studies have imaged in diastasis to minimize strain effects [37]. Methods exist for correcting the measured diffusion tensor for the effects of strain, using measured strain data [35], [36]. These methods are based on the simplified assumption that the heart is a jelly-like medium without microscopic hindrances or restrictions, and work has suggested that orientations obtained from the uncorrected strain tensor are closer to equivalent arrested heart data than the corrected data [38]. Until the effects of strain on diffusion measured within a complex microstructural environment can be quantified, we propose that the potential confounding effects of strain are acknowledged in published work using STEAM.

***Recommendation*** – The STEAM approach does not require specialized magnetic resonance imaging (MRI) gradient hardware. STEAM cDWI typically uses multiple signal averages (breath-holds) and its clinical application can be challenging, especially in patients with difficulties in holding the breath. STEAM should be used for cardiac DWI when a multiple breath-hold protocol is acceptable and/or imaging will be repeated at multiple phases of the cardiac cycle.

##### 3.3.1.2 Spin echo

Due to cardiac deformation, the monopolar SE diffusion-encoding approach leads to strong intravoxel dephasing and profound signal attenuation. For this reason, a conventional SE EPI monopolar DWI sequence (even with ECG triggering) is wholly inadequate for cardiac DWI [29]. An motion compensated spin echo (MCSE) is needed for cDWI [39].

*Gradient moment nulling* – Diffusion-encoding waveforms with nulled first (M1) [9], [40], and second (M2) [41], [42], [43], gradient moments offset the effects of intravoxel velocity and acceleration gradients. Remarkably, late systole is the cardiac phase in which the heart empirically most closely approximates the requirements of constant velocity and acceleration required by M1M2 gradient nulling.

*Spin echo advantages* – For the MCSE approach, the diffusion encoding and the signal collection (imaging) are realized in a single heartbeat. The SE approach has higher acquisition efficiency compared to STEAM and, owing to the improved SNR efficiency of SEs compared to stimulated echoes, affords additional SNR advantages. The MCSE approach is also relatively insensitive to respiratory-related motion, which allows free-breathing acquisitions with [44] or without navigators [43]. Heart rate variability also has a limited effect, compared to STEAM, and does not require b-value correction.

*Spin echo limitations* – MCSE DWI accords with intermediate TEs (70–80 ms) for moderate b-values (250–350 s/mm^2^) when using commodity gradient hardware (45 mT/m, 150 T/m/s). Note, Eq. (2) does not hold for M1M2-compensated waveforms. Owing to the moderate T2 of the heart (∼50 ms), it is difficult to use higher b-values because the required increase in TE results in substantial SNR reductions. For this reason, the MCSE approach substantially benefits from the highest available gradient performance or time-optimal gradient waveform design methods. Commercial systems with 80 mT/m body gradients are available and a b-value of 500 s/mm [2] can be achieved on these systems with M1M2-compensated waveforms and a TE of <80 ms [43]. Variation in MCSE protocols between sites requires attention when comparing data, as variations in TE and b-value will alter diffusion metrics.

***Recommendation*** – MCSE cDWI is a common alternative to STEAM cDWI. The MCSE approach requires M1M2-compensation and performs best when using a late-systolic TD and a high-performance gradient system (>50 mT/m and >100 T/m/s). The MCSE approach enables SNR-efficient, free-breathing acquisition of cardiac cDWI and cDTI during late systole.

### 3.4 Image acquisition

*Echoplanar imaging* – Both STEAM- and MCSE-based diffusion sequences typically use cardiac-triggered single-shot EPI. Rapid imaging minimizes motion-related blurring. The efficiency of single-shot imaging is important to allow acquisition of a sufficient number of diffusion-encoding directions for tensor reconstruction. Signal averaging is typically required to achieve adequate SNR. The use of segmented or multi-shot EPI remains very challenging for cardiac DWI, due to beat-to-beat differences in motion-induced image phase, which result in aliasing artifacts.

*EPI artifacts* – EPI readouts are prone to artifacts caused by several sources, including fat, off-resonance, T2* decay, and eddy currents [30]. Pre-excitation fat suppression methods are generally adequate for managing fat artifacts. So-called “Nyquist ghosts” common in EPI, due to misalignment of forward and reverse echoes, can be effectively corrected for using separately acquired reference data. Off-resonance effects contribute to image distortion and T2* decay leads to blurring and signal attenuation. These artifacts are minimized by using the shortest possible TE and EPI readout that maximizes the k-space traversal speed in the phase-encode direction [45], including via ramp-sampling and parallel imaging. The EPI readout duration can be further shortened by reducing the phase field of view (FOV) below the size of the imaged body. This so-called reduced-FOV approach is enabled by making one of the multiple RF pulses used to create the spin or stimulated echo slice-selective in the phase-encode direction [46]. TEs can be significantly reduced by using partial-Fourier methods, but this can result in a reduced effective spatial resolution, due to lower effective sampling densities at the margins of k-space, and motion-related signal loss can occur because the central k-space data can be shifted out of the sampled k-space region [47].

*Spatial resolution –* Typical in-plane spatial resolution is 2.5 × 2.5 mm to 3.0 × 3.0 mm, unless SNR constrained. Slice thicknesses are typically 6–10 mm. High in-plane spatial resolution (<2 mm in-plane) is desirable for resolving transmural variations in microstructure and thinner cardiac structures, and for detecting focal lesions. Higher spatial resolution reduces intravoxel deformation-related signal loss [47],[40] because the intravoxel phase dispersion caused by non-compensated motion during diffusion encoding decreases with voxel size. However, higher in-plane spatial resolution leads to lower SNR, owing to smaller voxel size and longer TEs. Compensating for poor SNR requires the acquisition of additional images, to maintain sufficient SNR to minimize measurement bias [48], [49].

*Advanced acceleration methods –* Slice interleaving increases SNR efficiency, but is difficult to combine with reduced FOV approaches because conventional RF pulses saturate or invert the magnetization outside the current slice. Simultaneous multi-slice (SMS) acquisitions efficiently excite multiple slices and resolve the overlapping images using information from multiple receiver coils [50], [51], but cardiac SMS remains challenging, owing to the heart’s comparatively small size in the slice-select direction.

*Non-Cartesian imaging* – Spiral readouts sample k-space efficiently and enable a very short TE because the central k-space data are collected first. However, spiral data are sensitive to off-resonance effects (blurring), T2* decay (blurring), and trajectory errors (image artifacts) [52]. While a number of studies have demonstrated spiral cardiac DWI [53], [54], [55], spiral acquisitions remain a challenging area for research [54].

***Recommendations*** – Both STEAM and MCSE approaches to cDWI should use single-shot EPI to acquire ∼2.5 × 2.5 mm to 3.0 × 3.0 mm in-plane resolution with ∼8 mm slice-thickness. This is typically implemented with full-Fourier imaging and rate-two parallel imaging acceleration, but partial-Fourier techniques can also be valuable. A reduced FOV approach can help, if available.

### 3.5 Hardware considerations

High-performance MRI systems are routinely used for cardiac MRI exams and, with the appropriate pulse sequences and protocols, are well suited to the acquisition of cardiac DWI. Herein, we discuss MRI system considerations as they apply to cardiac DWI.

*Main magnetic field (B0)* – cDWI has been reported at 1.5T, but most of the reported work is at 3T. This partly reflects the increasingly broad installation base of 3T MRI systems and the potential for higher SNR. Commercial 3T systems also frequently have available higher performance gradient systems, owing to market demands that are largely driven by neuro and musculoskeletal applications. However, 1.5T MRI can have better field homogeneity and exhibit longer T2/T2*, which can mitigate susceptibility and distortion artifacts for EPI data acquisition. Magnetic field shimming (specifically localized to a tight box around the RV and LV) is an important component of a robust cardiac DWI protocol.

*Radiofrequency (RF or B1)* – In general, there are no specific RF considerations for cDWI, as the most common sequence variants (STEAM and MCSE) are not high Specific Absorption Rate sequences.

*Gradient hardware* – Depending on the specific cardiac DWI sequence, the specific gradient hardware plays a greater or lesser importance in the final image quality. High-performance gradient systems (>50 mT/m and >100 T/m/s) are important to achieve intermediate b-values (200–450 s/mm^2^) in short diffusion-encoding durations (<50 ms) for MCSE-based protocols. High-performance gradient systems are also important for very short (<1 ms) echo spacing in EPI readouts, to limit distortion and ghosting artifacts. The availability of 80 mT/m gradient systems with slew rates of 200 T/m/s has proven effective for cDWI with MCSE-EPI [41], [43]. Commercial systems with wide bores and gradients of 60 mT/m are now available, but the experience with cDTI on these systems is limited. In addition, commercial systems with 150 mT/m or more whole-body gradients are now available and could facilitate substantial reductions in TE- with SE-based sequences. In general, the STEAM approach does not require (nor benefit from) high-performance gradients for improved diffusion encoding.

*Physiological monitoring* – Cardiac DWI requires synchronization with the cardiac cycle, which is best achieved via ECG triggering. Pulse oximetry triggering generally does not perform well, owing to the lower timing precision and the subject-dependent delay between the detected signal and cardiac activity. For the SE approach, TD sweeps [44] have been shown to be effective in identifying the cardiac phase with best diffusion image quality. The STEAM approach is routinely acquired using repeated breath-holds, but the MCSE-EPI approach can be acquired during free-breathing with prospective (navigator) or retrospective respiratory gating.

***Recommendation*** – Most cardiac DWI reports use 3T MRI with 16- to 32-channel receiver coils and ECG triggering. If the STEAM approach is used, then the gradient hardware specifications are not an essential consideration. If the MCSE approach is being used, then high-performance gradients (≥80 mT/m and ≥100 T/m/s) have an advantage and should be used, if available.

### 3.6 Acquisition protocols

*Acquisition duration, averages, and directions* – Cardiac DTI requires a combination of multiple b-values (b_val_ ≥ 2), with multiple diffusion-encoding directions (N_dir_ ≥ 6) at the higher b-value, resulting in ≥7 (N_combo_) images per slice. Signal averaging (N_avg_ ≥ 1) is also typically required to produce sufficient SNR. Consequently, if each image is acquired over t_R-R_, the total acquisition duration per slice, equivalent to t_R-R_·N_combo_·N_avg_, exceeds 14 heartbeats for STEAM and 7 heartbeats for MCSE. More typically, many averages (N_avg_ ≥ 4) are required for adequate SNR. Many directions (N_dir_ ≥ 10) are also commonly used, to increase angular resolution, minimize the chance of losing a direction to signal drop-out, and improve tensor SNR. In combination, this necessitates repeated breath-holds or free-breathing approaches. The best combination of N_dir_ and N_avg_ remains an area of investigation [56], [57].

*b-value* – Optimal choice of the two b-values maximizes the ratio between the diffusion-weighted signal loss and noise, and improves tensor precision. However, with very high b-values (values depend on SNR), the signal approaches the background noise, with resulting underestimations of FA and MD [48], [49]. Perfusion can also result in a diffusion-like signal loss [58], [59], [60]. The active nature of perfusion means that the perfusion-related signal decays rapidly at low b-values. Therefore, a non-zero lower b-value (b_min_ >0 s/mm^2^) should be used to minimize the contribution of perfusion to diffusion measurements [58], [61], which also reduces the ventricular blood signal in SE sequences.

STEAM sequences typically use a high b-value, ranging from 350–500 s/mm^2^ [35], [61], and SE methods typically use 250–500 s/mm^2^ [41], [43]. A study using STEAM found an optimal pairing of 150 and 750 s/mm^2^ [61], although this is likely to vary with SNR. For SE methods, the optimization is more difficult, because large b-values also substantially increase TEs and will vary based on the available gradient strength.

***Recommendation***: To enable meaningful comparison between studies, we recommend that all cardiac diffusion studies should consistently report the following acquisition protocol parameters:

• Diffusion—number of non- (or low-) diffusion-weighted images and b-value, number of diffusion-encoding directions and b-value, gradient (maximum amplitude and slew rate) parameters.
• Motion compensation—name and reference (e.g., monopolar STEAM over two heartbeats, MCSE, etc.) and/or pulse sequence timing diagram.
• k-space—trajectory details, partial/full Fourier, TE, echo spacing, readout bandwidth.
• Spatial resolution—acquisition matrix size, FOV, slice thickness, image interpolation (if any).
• Acceleration methods—including parallel imaging, partial Fourier, and reduced FOV techniques.
• Fat suppression technique.
• Timing—repetition time (TR in ms or number of RR intervals), breath-hold, and/or total acquisition duration per slice, number of signal averages; TD as percentage of the RR interval
• Radiofrequency (RF)—STEAM or MCSE, flip angles, and RF-pulse type.

### 3.7 Managing artifacts

*Motion* – Patient movement, and the motion of the heart with the respiratory and cardiac cycles, can result in mis-registration between diffusion-encoded images within and between slices, blurring, and ghosting. Furthermore, the diffusion-encoding gradients make these sequences particularly vulnerable to profound artifactual signal loss. The effects of respiratory motion are best minimized using breath-holding. MCSE acquisitions can be gated using diaphragmatic navigator gating, with or without slice tracking [62], but this is challenging to perform with STEAM, due to three RF pulses being applied over two heartbeats [33]. Retrospective respiratory gating can also be used with SE cDTI, but may be less effective at the apex of the left ventricle [43].

*Perfusion Effects* – Diffusion imaging is sensitive to any intravoxel incoherent motion (IVIM) (diffusion, perfusion, deformation). Perfusion within the myocardial capillaries can contribute to the apparent measured diffusivities. The apparent diffusion coefficient of the perfusing water molecules in the blood (known as a pseudo-diffusion coefficient in IVIM studies) is assumed to be higher than the apparent diffusion coefficient of the diffusing water molecules [62]. As a result, the signal from the perfusing water molecules decays quickly at low b-values (∼50 s/mm^2^), leaving predominantly diffusion-related signal loss at higher b-values (>100 s/mm^2^) and effectively eliminating perfusion effects [61], [63]. Optimization of b-values is required to avoid microvascular perfusion effects and also helps attenuate residual ventricular blood signal in the “non”-diffusion-weighted image.

*Blood Signal Artifacts –* Ventricular blood signal is well suppressed in STEAM sequences (even with very low b-values), due to the outflow of blood during the long TM (∆). Motion-compensated SE images may show residual ventricular blood signals that can complicate segmenting the myocardium and contribute to partial volume effects at the endocardial border.

*Fat Artifacts* – Fat is off-resonance and appears shifted along the phase-encode direction (very low bandwidth) in EPI acquisitions and is smeared in-plane in spiral acquisitions. Fat suppression is thus essential and can be achieved via fat saturation or water-selective excitation pulses. Water-selective excitation pulses are commonly used in MCSE acquisitions, due to their robust nature. Due to the short T1 of fat, its signal relaxes quickly during the long TM of the STEAM sequences. Consequently, the STEAM sequence has intrinsic fat suppression, because fat contributes little to the stimulated echo.

*Off-resonance Artifacts* – Off-resonance-related distortion and blurring occur in the phase-encode direction in EPI (all directions in spiral), due to local changes in magnetic susceptibility. This is most evident in the inferolateral LV free wall in the proximity of cardiac veins and at air-tissue interfaces. The susceptibility gradient scales linearly with the main magnetic field, hence off-resonance distortions are more severe at 3T than 1.5T. Including B0 maps into the image reconstruction or increased phase-encode bandwidth, by undersampling, both help. These off-resonance artifacts can be reduced by using local or cardiac-specific shimming methods.

*Diffusion Gradient-Induced Artifacts* – The diffusion-encoding gradients can cause eddy current-related distortions in the images that vary between diffusion-encoding directions and b-values [64]. Correction can be performed in the pulse sequence [65] or via a postprocessing step .[55] For the use of diffusion gradients that are not balanced around the echo pulse in MCSE imaging (or potentially the second and third RF pulse in STEAM imaging), concomitant field effects must be considered [65].

***Recommendation***: cDWI sequences should employ ECG triggering and respiratory motion management. Motion-compensated diffusion gradients are required, and water-only excitation (to limit fat artifacts) may be useful for SE approaches. Non-zero low b-value images help suppress perfusion and intraventricular blood artifacts. Specialized shimming approaches should be used to limit off-resonance image distortion.

### 3.8 Optimization of image quality

Analogous to balanced steady-state free precession (bSSFP) imaging, cDWI relies completely on endogenous contrast and the acquisition can, therefore, be repeated at the scanner if artifacts are present. However, unlike bSSFP, where off-resonance artifacts can be easily recognized and addressed with frequency scouting, no single and rapid metric of quality is available for cDTI. In addition, cDTI is vulnerable to many types of artifacts. Therefore, it is highly advisable for those new to the field to partner with a site with more experience. An early decision will often involve which type of sequence to use, based largely on the availability of systems with gradients >80 mT/m. The user should be aware that normal values of MD and FA will differ between diffusion-encoded spin and stimulated echo sequences [66], and use reference values appropriate for the type of sequence being implemented (Table 4). The impact of age and gender on the normal range of DTI-derived metrics will need to be further studied.

**Table 4:** Approximate normal values [33], [34], [44], [54], [65], [67], [68], [69], [70], [71], [72], [73].

	Sequence	Cardiac phase	Approximately normal range mean ± SD (typical range)	Comment
MD (×10^−3^ mm^2^/s)	STEAM	Systole and/or diastole	1.03 ± 0.11 (0.8–1.2)	MD with STEAM is lower than SE
FA	STEAM	Systole	0.47 ± 0.04 (0.43–0.51)	FA with STEAM is higher than SE
FA	STEAM	Diastole	0.58 ± 0.04 (0.54–0.62)	
E2A°	STEAM	Diastole	13° ± 6° (7–20°)	Absolute values of sheet angle used
E2A°	STEAM	End systole	62° ± 5° (51–67°)	
MD (×10^−3^ mm^2^/s)	MCSE	Systole	1.45 ± 0.23 (1.22–1.68)	MD with SE is higher than STEAM
FA	MCSE	Systole	0.36 ± 0.04 (0.22–0.58)	FA with SE is lower than STEAM
E2A° (SE)	MCSE	Mid systole	38° ± 8° (34°–47°)	Absolute values of sheet angle used
HA range	STEAM/MSCE	-	95–110° (∼+50° to ∼−50°)	With trabeculations excluded (endo to epicardium)
HA transmurality	STEAM/MSCE	-	−1 ± 0.06°/% thickness	Slope is negative (endo to epicardium)
E2A° mobility	STEAM/MSCE	-	45° (39°–50°)	Radial re-orientation of sheets in systole

*SD* standard deviation, *MD* mean diffusivity, *STEAM* stimulated echo acquisition mode, *SE* spin echo, *FA* fractional anisotropy, *E2A* absolute sheetlet angle, *MCSE* motion-compensated spin echo, *IQR* interquartile range, *HA* helix angle

While no single scheme to ensure quality control can be prescribed, we describe an approach below that could be used in a quality-control cohort of 20 healthy volunteers. When acquiring and analyzing these datasets, the quality of the raw images, and scalar maps of MD, FA, and HA maps should be assessed. Specific attention should be paid to the identification of artifacts due to incomplete fat suppression, motion, flow, the EPI readout, and signal loss due to susceptibility effects. The user should be able to correctly predict and measure the expected drop in signal when the high b-value images are acquired. Experienced readers should also be able to identify the tri-layered (i.e., epi-mid-endocardial) presence of diffusion contrast in the myocardium on some of the high b-value images and understand when/where to expect this. The user should be familiar with the upper limits of MD and FA in the myocardium [66], and the expected differences in these values with stimulated and SE acquisitions. The new user should further be familiar with the transition of cardiomyocyte orientation (HA) across the human myocardium [43], [74], and be able to identify when this is falling outside of the normal physiological range (too low or too high).

For those novice users unable to partner with an experienced center, we provide a heuristic approach, with the potential to identify/eliminate common artifacts, as the novice user gains experience with DTI of the heart. This should be viewed as a rough and experimental guide and not as a definitive algorithm.

**Recommendations**

• Twenty normal volunteers should be imaged in each center, to establish the technique, define average normal values, and ensure robust image quality. Additional volunteers may be needed as the technique matures and becomes clinically mainstream.
• All images should be acquired with validated cardiac-DTI sequences with a tightly controlled protocol. Sequences developed for other body parts, such as the brain, should never be used.
• Draw an ROI in septum of low b-value and high b-value images. The reduction in signal should scale roughly as e^-bD^ (Eq. (1)). In practice, a signal reduction of 30%–50% usually strikes a balance between sufficient diffusion contrast and adequate SNR in the high b-value images.
• Acquire a breath-hold single short-axis slice DTI image at the mid-ventricular level in mid-end systole perpendicular to the long axis. For initial quality control, the use of six diffusion-encoding directions is recommended. Repeat the acquisition to acquire six to eight averages (using STEAM, this will add up to eight breath-holds to the standard CMR examination). cDTI with free-breathing can also be similarly acquired with the SE approach.
• Use of the scanner’s inline software to assess MD and FA is not recommended as too noisy, lacking image co-registration and lacking rejection of corrupted images.
• Further postprocessing and analysis of the images require dedicated software.
• Once adequate image quality has been established, definitive imaging should be performed with six or more diffusion-encoding directions.
• STEAM acquisitions must be acquired during a breath-hold. SE acquisitions can be acquired with a breath-hold or with either prospective or retrospective respiratory gating.
• Prospective cardiac triggering should be used in all cases.

If the images are suboptimal:

• Imaging the heart in mid to late systole is typical and this requires adjusting the TD. A “trigger delay scout” sequence, in which a single high b-value image is repeatedly acquired at varying delays after the R wave, can also be used to identify the optimal trigger time.
• If image quality remains poor, optimize the shim and minimize the TE; perhaps acquire data with a lower b-value for testing purposes.
• If the subject has an irregular cardiac rhythm, change to an SE acquisition.
• If the subject cannot hold their breath, then use a free-breathing SE sequence.

A table of approximate normal values for commonly measured parameters, acquired in vivo in healthy subjects, is provided (Table 4).

Helix angle transmurality (HAT) is measured as the linear slope in HA from endocardium to epicardium, and can be reported as a negative or absolute value, either in units of ˚/mm or normalized to wall thickness to give ˚/%. The method used should be clearly defined in all publications. In healthy subjects, artificially high MD values, low FA values, and a low HA range/transmurality should raise suspicion for poor image quality and artifacts.

Full anatomical coverage of the heart without any slice gaps is ideal but is not practical in most clinical settings and research settings. It is recognized that the researcher/clinician is often forced to reduce coverage, but this should be done thoughtfully and in the context of the disease being studied and the question being addressed. No single recommendation for the acceptable number of slices, their location, and the gap between them can be made at this time. This should be tailored to the pathology being studied and the question being asked, and should be clearly justified in the study design. In general, confidence in a study’s conclusions will be increased if more than 1 slice is acquired, and this is encouraged. To date, no consistent differences in MD, FA, HAT, and E2A have been documented between short-axis slices at the base, mid-LV, and apex, although this should be further studied.

### 3.9 Postprocessing display and reporting

#### 3.9.1 General postprocessing workflow for cDTI

**Fig. 3:**
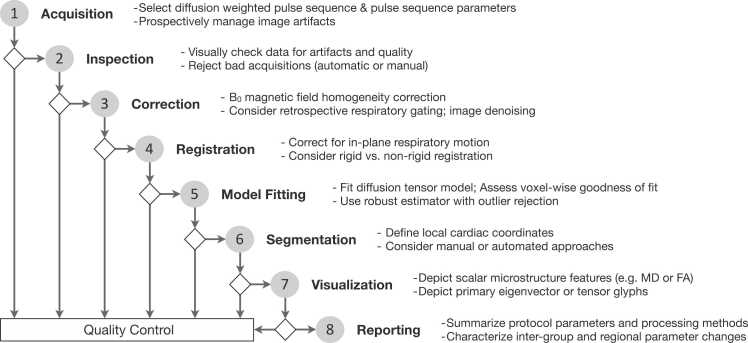
**Suggested cDTI postprocessing workflow.** Summary of the steps needed for the accurate postprocessing of cDTI images. *cDTI* cardiac diffusion tensor imaging, *MD* mean diffusivity, *FA* fractional anisotropy

Postprocessing encompasses all the calculations performed on the diffusion-weighted images. Postprocessing workflows have been developed in-house by different groups. There are currently no standard tools to analyze cDTI data, but consensus exists in several areas. The postprocessing pipeline, as described in Fig. 3, provides a typical approach.

*Data inspection:* In addition to physiological noise from respiratory and cardiac motion, diffusion quantification with MRI inherently produces low-SNR images. To mitigate this, it is common to acquire either multiple repetitions of the same diffusion images and/or a large number of diffusion-encoding directions. The acquired data need to be inspected manually (i.e., visual inspection) or with automated methods suitable for finding outliers (corrupted images), including machine-learning-based approaches [75]. Signal outlier detection methods during tensor fitting can also be used, such as the Geman–McLure M-estimator [76]. No single data-curating approach or technique has been shown to be superior at the present time.

*Corrections:* Noise-reduction strategies can exploit the availability of multiple repetitions, by using principal components analysis [77], [78], [79] or machine-learning-based approaches [80]. Retrospective gating of free-breathing diffusion-encoded SE data can be performed using a low-rank tensor multitasking-based approach [43] and principle component analysis-based techniques [78]. At present, no technique to perform retrospective cardiac gating of diffusion-encoded images has been broadly used. The most common metrics for noise-reduction assessment are SNR and contrast-to-noise ratio. Image distortion due to the EPI readout can be corrected by various methods, such as the acquisition of blip-up and blip-down EPI data [81], which has been used in other organ systems.

*Registration*: In general, there are two types of image registration techniques, those based on rigid transformations (rotations and/or translations) and non-rigid transformations (elastic, spline-based, point set registration, etc [82], [83]). Non-rigid methods can be computationally intensive and may require expert segmentation or landmarks to accelerate, guide, and improve the precision of the correction. The selection and tuning of the motion correction scheme can depend on the quality of the images and deformation of the structure of interest [51]. Metrics of the quality of image registration usually rely on segmentation, such as DICE similarity coefficient or Hausdorff distance, or the analysis of a line profile in temporal or diffusion-gradient dimensions. Currently, there is no standard image registration scheme and several key components of the method (technique, metric, optimizer, landmarks, etc.) are generally bespoke.

*Model-Fitting /Diffusion Tensor Estimation and Tensor Parameters:* Multiple approaches are available for reconstructing diffusion tensors from the diffusion-weighted images, ranging from a basic linear least-square to more complex iterative non-linear algorithms. For STEAM sequences, it is recommended not to average repetitions of the same b-value and diffusion direction, but instead use an overdetermined system of equations for tensor fitting. This approach allows for the correction of individual b-values due to RR interval variation.

Several different parameters are then derived from the tensor’s eigensystem. These can be divided in two different categories: rotational-invariant measures related to tensor shape determined by the eigenvalues (λ_1_, λ_2,_ and λ_3_), and tensor orientation measures determined by the eigenvectors (e_1_, e_2_, and e_3_) (Fig. 1).

**Fig. 4:**
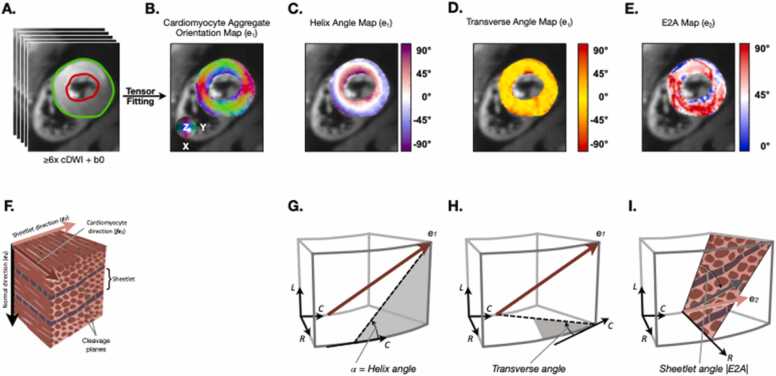
**Coordinate system used for derivation of helix, transverse and sheetlet angles in the heart.** (A) Segmentation of the myocardium. (B) Direction of primary eigenvector in each voxel, depicted using a conventional Cartesian coordinate system. (C-E) The angles of the primary and secondary eigenvectors with the local cardiac coordinate system can be used to derive the helix, transverse, and sheetlet angles. (F) Schematic of myocardial microstructure. (G-I) Coordinate system based on the radial (R), circumferential (C), and longitudinal (L) vectors in the heart. (G) The helix angle is calculated by projecting the primary eigenvector (brown) onto the epicardial tangent (L-C) plane of the LV. The helix angle is defined as the angle that this projection makes with the plane bounded by the local radial and circumferential vectors (short-axis plane). (H) Likewise, the transverse angle is defined by projecting the primary eigenvector onto the R-C (short-axis) plane and measuring the angle that this projection makes with the plane defined by the local circumferential and longitudinal vectors (epicardial tangent plane). (I) The absolute sheetlet angle (E2A) is defined as the angle between the projection of e2 into the radial-cross-myocyte plane and the cross-myocyte direction, where the cross-myocyte direction is perpendicular to e1proj within the local L-C (epicardial tangent) plane, and e1proj is e1 projected on the local L-C (epicardial tangent) plane. *cDWI* cardiac diffusion-weighted imaging

The tensor orientation measures are normally described in relation to the local wall coordinates, which consist of circumferential, longitudinal, and radial directions that follow the curvature of the heart wall [84]. Therefore, it is common to transform diffusion tensors from the raw diffusion image coordinates to a cardiac coordinate system (specific to an individual’s heart) before deriving metrics of local myocyte orientation. These include the HA and transverse angle (the magnitude of which is much smaller than the HA), which are defined as angles made between the primary eigenvector in a voxel and the radial, circumferential, and longitudinal planes. By convention, the projection of the eigenvector onto the local wall coordinate system is used to calculate these angles (Fig. 4) [84].

A more intuitive definition of the helix, transverse, and sheetlet angles is provided in Table 5.

**Table 5:** Intuitive definitions of helix, transverse, and sheetlet angles.

	Technical definition	Intuitive definition
Helix angle	Angle between projection of primary eigenvector (e1) onto epicardial plane and local R-C (short-axis) plane	Angle with which primary eigenvector (cardiomyocyte) tilts out of short-axis plane toward either the apex or base
Transverse angle	Angle between projection of primary eigenvector (e1proj) onto short-axis plane and local (L-C) epicardial tangent plane	Angle with which primary eigenvector (cardiomyocyte) tilts from epicardial tangent plane toward LV cavity
Sheetlet angle	Angle between the projection of e2 into the radial-cross-myocyte plane and the cross-myocyte direction (direction is perpendicular to e1proj within the local L-C, epicardial tangent, plane	Orientation of myocardial sheetlets (parallel to perpendicular) with respect to local epicardial tangent plane. Low sheet angle (diastole) = more parallel to epicardial tangent plane

LV *left ventricle*

*Segmentation*: Local cardiac coordinates are normally defined by the segmentation of anatomical structures of interest. After delineation of the short-axis LV endocardial and epicardial contours, a cardiac-specific coordinate system can be defined for which the radial direction is perpendicular to the contour, the circumferential direction is tangent to the contour, and the longitudinal axis is assumed as perpendicular to the short-axis slice. This segmentation may be manual, interactive (semi-supervised), or automatic (unsupervised). Once the measures of interest have been extracted from the pixel-wise diffusion tensor, several subsequent segmentation options are available: 1) the entire LV myocardium is segmented to establish an average value, 2) the myocardium is divided into 16 segments, as defined by the American Heart Association guidelines, or 3) ROIs are drawn in regions of pathology and healthy (remote) tissue for comparison. While no systematic study of ROI size and location has been performed, it is reasonable to follow the guidelines used for other mapping techniques, such as relaxation rate mapping, by measuring parameters of interest in the interventricular septum. We recommend, however, that the ROI encompass the entire thickness of the LV wall from endocardium to epicardium (with care taken to avoid blood-pool partial volume effects, trabeculation, and papillary muscle) unless strong justification related to a specific disease can be provided for a narrower ROI.

*Visualization:* Diffusion tensor results are wide-ranging, often conveying 3D information, and difficult to communicate to a non-expert readership. It is therefore important to display results in a clear and unambiguous way. Color maps beyond grayscale are normally used but it is important to consider two properties: first, the perceptual qualities of the color map (for example, color-blindness, perceptual uniformity of lightness); and second, the properties of the parameter shown (sequential, diverging, or cyclical) [85] For example, the HA values are cyclical, and therefore care must be taken to use a cyclical hue that wraps around ±90˚.

**Fig. 5:**
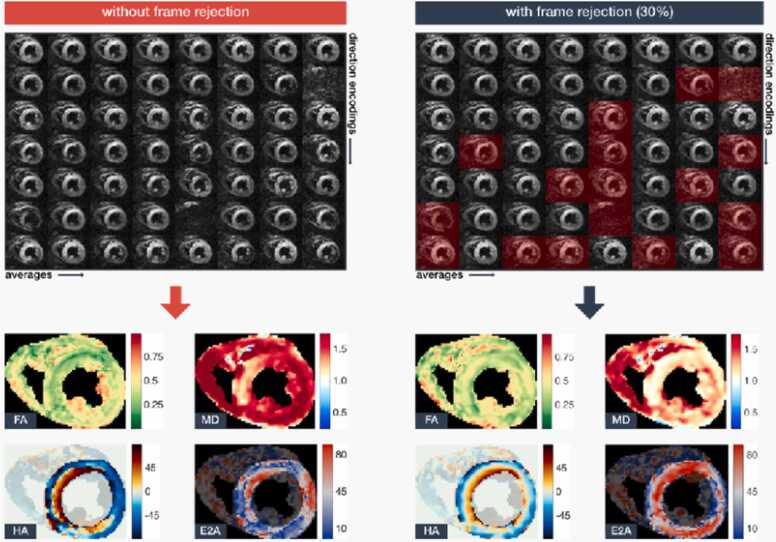
**Impact of image curation on the quality of cDTI parameter maps.** Top: cDTI data (cropped to the heart only): left: entire dataset used for tensor fitting; right: images corrupted with signal loss removed manually (red transparent mask) before tensor fitting. Bottom: respective tensor parameter map estimations showing: fractional anisotropy (FA) (unitless), mean diffusivity (MD) (×10^−3^ mm^2^/s), helix angle (HA) (˚), and absolute sheetlet angle (E2A) (˚). Data with outlier rejection has a lower MD, a higher FA, more transmurally-structured HA, and more uniform E2A, compared to data without outlier rejection. The images shown are STEAM breath-hold acquisitions in a healthy subject in a 3T Siemens (Skyra) scanner. *cDTI* cardiac diffusion tensor imaging, *STEAM* stimulated echo acquisition mode

Tensors and eigenvector fields can be represented with 3D glyphs of ellipsoids and thin cylinders, respectively. Even though the geometry of an ellipsoid is directly related to the tensors’ eigensystems, it can be difficult to interpret the ellipsoids’ shape from a 2D image. For this reason, ellipsoids are often replaced with superquadric glyphs, which are designed to improve tensor visualization [86]. 3D tractography provides an extremely useful tool to comprehensively visualize the orientation of cardiomyocytes over the entire heart [9], [19], [87], [88]. As discussed above, the generated tracts or streamlines are virtual and do not represent physical fibers or myofibers. Several metrics of microstructure can be derived from the tracts, but these remain investigational [11], [88]. Quantification of cardiomyocyte orientation should be performed using 2D HA maps with the following metrics: HA range (max to min) and HA slope in degrees per percent transmural thickness. This latter parameter is often referred to as HA transmurality or HAT (°/mm or °/% transmural thickness) [43], [74] (Fig. 5).

**Recommendations**

• Standardization of the postprocessing workflow is recommended; the pipeline should include all the routine steps as listed in Fig. 3. As before, new centers should partner with established sites with experience in postprocessing.

• Data inspection/quality control: remove cDWI data/images corrupted with artifacts and/or exhibiting motion-induced signal loss. Currently, there is, as yet, no consensus on how many repetitions can be removed without impacting the accuracy of the measurement; most centers would obtain more than six directions, in case one is corrupted, and more than four averages/repetitions.
• Registration: motion correction using at least rigid registration must be performed before any pixel-wise filter or model fitting.
• Model fitting: there is no consensus for modeling fitting, but linear and non-linear least-squares approaches are most common.
• Segmentation: a conservative approach to avoid blood-pool partial volume artifacts is recommended. Avoid trabeculations for transmural measurements.
• Visualization: it is important to display results in a clear and unambiguous way. Color bars should be included in all figures, with the scale clearly displayed.
• In addition to MD and FA, the commercial vendors should consider providing an option for inline HA calculation of cardiac DTI datasets.
• 3D tractography provides a valuable tool to visualize cardiomyocyte orientation over the entire heart. However, the quantification of cardiomyocyte orientation should be performed using 2D HA maps.

### 3.10 Scientific and clinical interpretation

DWI is uniquely sensitized to the presence and mobility of water in the different tissue compartments. In stationary tissues, advanced diffusion-encoding schemes can be used to resolve the displacement of water molecules within each tissue compartment, including the intracellular, extracellular, and intravascular spaces. cDTI provides a composite measure of water diffusion in all compartments, although the low b-value gradients should reduce/null the signal from the intravascular space. The primary eigenvector of diffusion in the heart reflects diffusion in both the extracellular and intracellular spaces. Intracellular restriction of diffusion is influenced in part by the dimensions/shape of the cardiomyocyte (where diffusion is known to be greater along the long axis of the cardiomyocytes compared to across their minor diameters) and by the arrangement of intracellular structures, such as sarcomeres and mitochondria.

cDWI using the IVIM approach in the heart has the potential to distinguish the motion of protons in the intravascular and extravascular spaces and, consequently, measure perfusion in the microcirculation without the need for an exogenous tracer [62] [58].

The diffusion coefficient (D) of water in a material is usually reported as an average value. This reflects a distribution of values, because individual protons will undergo a range of displacements due to diffusion over a given time [89]. Timing differences in the diffusion-encoding approach, combined with this phenomenon, likely underlie the difference in MD and FA values obtained with diffusion-encoded spin and stimulated echoes [34], [72], [89]. With the SE approach, the b-value (strength of diffusion encoding) is produced by the application of extremely strong gradients for a brief amount of time. This biases the measurement toward those protons with higher diffusivity, which can move substantially during the short time the diffusion gradients are applied. In contrast, the gradients applied during a stimulated echo sequence are significantly weaker, and the desired b-value (degree of diffusion encoding) relies heavily on the long TM in the sequence. The long TM in the stimulated echo formalism provides more opportunity for spins to encounter diffusion barriers, which will result in lower measured diffusivities with STEAM. The biophysical properties of the SE approach also make it more sensitive to rapid short-range diffusion, which reduces the differences between the primary, secondary, and tertiary eigenvalues. Consequently, FA values with the SE approach are approximately 30% lower than they are with the stimulated echo approach (Table 4) [34], [66], [72].

Preclinical and clinical studies using cardiac DTI have revealed some consistent patterns. MD increases in an acute injury, such as myocardial ischemia, and FA decreases [9], [13], [15], [88], [90], [91], [92], [93]. Depending on the specific pathophysiological process, an increase in MD may reveal an increase of the extracellular space (increased ECV), an increase in cellular size/swelling, or an increase in blood supply (high perfusion). The ECV, in turn, can be increased due to rupture of the cardiomyocyte cell membrane (necrosis) or due to fibrosis, protein deposition (amyloid), and inflammatory conditions, such as sarcoidosis or myocarditis. At the present time, no pattern of diffusivity specific for any of the above processes has been described. An increase in diffusivity should therefore be interpreted in its clinical context and in conjunction with other findings, such as those obtained from maps of relaxation rate (T1, T2), and LGE imaging patterns.

A reduction in the FA of the myocardium can be seen in acute and chronic ischemic injury [9], [13], [15], [88], [90], [91], [92], [93], HCM [37], [94], and infiltrative conditions (e.g., amyloid) [71], [95]. Likewise, changes in the cardiomyocyte orientation or HA have been observed in acute and chronic ischemic injury [9], [16], [88], [90], [96]. Typical patterns include a loss of cardiomyocytes with positive (right-handed) HAs, a reduction in HAT, and a reduction in orientation coherence on tractography [9], [16], [88], [90], [96]. In healed infarcts, the HA pattern can also be influenced by the presence of residual cardiomyocytes in the infarct and the microstructural pattern of the collagen fibrils in the scar [97], [98]. The natural history of HA in non-ischemic cardiomyopathy has been less extensively studied. Changes in cardiomyocyte HA have been detected in some [71], but not all [12], non-ischemic cardiomyopathies, and definitive observations on HA in non-ischemic cardiomyopathy cannot be made at this time.

Alterations in sheetlet orientation (E2A) have been described in ischemic [96], [99], hypertrophic [12], [70], [94], [100], infiltrative [71], [95], and dilated cardiomyopathy [12], [101]. In general, those conditions, such as HCM, that are characterized by hyperdynamic contraction, have higher E2A values in systole relative to healthy controls [12], [70], [94]. Conversely, dilated cardiomyopathy is characterized by lower E2A values in systole relative to healthy controls [12], [101]. Furthermore, conditions associated with impaired diastolic relaxation, such as HCM [12], [70], [94] and amyloid [71], [95], have abnormally high E2A values in diastole. E2A mobility can be reduced due to either reduced myocardial contraction or relaxation.

cDTI is ideally suited to longitudinal studies, since it does not require ionizing radiation or the administration of exogenous contrast agents. Serial imaging in recovered dilated cardiomyopathy, for instance, has shown that abnormalities in myocardial microstructure do not resolve fully, even when the ejection fraction recovers [102]. Likewise, serial cDTI studies in subjects with MI have provided important insights into the relationship between myocardial microstructure and cardiac remodeling [96], [103].

Much of the work with cDTI remains experimental and preliminary. In the clinical arena, the field is still in its nascency, and a critical mass of studies has only been performed in two conditions, namely MI and HCM.

In acute MI, the following changes have been reported: an increase in MD in the infarcted region [15], [91], [93], [96], [103], a decrease in FA, and reduced E2A mobility in systole [15], [103].[93] Changes in E2A compatible with steeper sheetlet orientation have also been reported [15], [102].[93] After healing, the chronic infarct region is characterized by diffuse fibrosis and microstructural remodeling, including cardiomyocyte disorganization [88].[13] The following changes have been reported in chronic infarcts: an increase in MD [88], [103], a further decrease in FA, and changes in HA [15], [96], [103]. In vivo cDTI may provide new mechanistic insights into adverse left ventricular remodeling post MI. Initial evidence suggests that low FA and systolic E2A (as reflective of sheetlet angle) may be independently associated with long-term adverse remodeling .[96] While all of these initial results are encouraging, further studies and multi-center trials will be needed to reproduce these findings and determine their reproducibility.

HCM presents a wide range of underlying alterations in the cardiac structure and function and has been of major interest to the diffusion research community. HCM induces a loss of cardiomyocyte organization, compared to normal hearts, and can also be characterized by local fibrosis. Studies using cDTI have yielded inconsistent changes in HA, even in areas of the myocardium that are highly hypertrophied and show late gadolinium enhancement. However, alterations in E2A mobility from diastole to systole [12], [100] have been consistently detected. Notably, E2A values in HCM are elevated in diastole (∼50˚), compared to healthy volunteers (∼10˚), which suggests E2A could be used as a marker of disease in HCM. Initial data show the potential emerging clinical role of cDTI in HCM risk stratification. Specifically, changes in DTI biomarkers in HCM have been associated with early changes in perfusion and could be early markers of the disease [69], [70]. In addition, a reduction in FA in the mid-septum of patients with HCM has been shown to correlate independently with the presence of ventricular arrhythmias [37] and could become a marker of risk for sudden cardiac death.

The added value for pure diagnosis and/or monitoring with cDTI, compared to existing techniques, remains to be fully demonstrated. More efficient multi-slice and free-breathing acquisition strategies may ease applicability and dissemination. In its current state, however, cDTI is still used predominantly as a research tool to understand and phenotype cardiac pathologies. Firm recommendations on the use of cDTI in the clinical area can, therefore, not yet be made at this time.

**Recommendations**

• Those using cDTI should be familiar with the biophysical basis for pathophysiological changes in diffusivities, differences in diffusion sensitivity obtained with diffusion-encoded SE versus stimulated echo, and how this affects MD and FA values obtained with the two techniques.
• Additional clinical studies in ischemic heart disease, HCM, non-ischemic cardiomyopathy, and the full range of cardiovascular conditions are strongly encouraged.
• Acquisition and analysis techniques should be developed by the commercial vendors as “works in progress” sequences, that will allow multi-center studies to be performed using standardized techniques and methodologies.
• cDWI should ideally not be performed alone, or interpreted in isolation, but rather in a combination with bSSFP cine, strain imaging, T1/T2 mapping and, when appropriate, LGE and ECV. The interpretation of changes in diffusivity, anisotropy, and cellular microstructure should be interpreted in the specific clinical context and in conjunction with other metrics provided by CMR.

### 3.11 Rigor and validation

Independent verification of the myocardial microstructure derived from cDTI has been an active research area for more than two decades. There are three aspects that underpin and substantiate our recommendations.

(i) Preclinical studies. Numerous studies have compared cardiomyocyte orientation patterns obtained by ex vivo DTI with histology [8], [104].[84] The correlations have been very strong in both fresh and fixed hearts. There is broad consensus that tissue fixation changes the eigenvalues (and hence MD and FA) of the myocardium significantly, but does not change the eigenvectors or preferential directions of diffusion. The principal limitation of these ex vivo studies, however, is the absence of any load on the heart or perfusion during the diffusion experiment, and in some cases no control over systolic versus diastolic arrest. The use of perfused hearts in multi-state sequential experiments has addressed some of these concerns and has confirmed the high correlation between cardiomyocyte orientation by DTI and histology. Langendorff-perfused rat hearts were imaged with an SE approach, first in their slack state during cardioplegic arrest, then during lithium-induced contracture [100], [105]. Both cardiomyocyte and sheetlet structures were characterized and validated against histology. The transmural variation in HA was seen to undergo small changes from relaxed to contracted states, while the sheetlet structure re-oriented substantially between relaxed and contracted states [106]. Similar conclusions were reached in an in vivo study in swine, where STEAM cDTI was acquired at several cardiac phases, followed by in situ and ex vivo scans. cDTI findings compared well with co­registered histology in both relaxed and contracted states in this swine model. The microstructural changes from relaxed state to contraction were consistently observed under all experimental conditions and correlated with histology [12]. In vivo cDTI was also performed in a swine model, using motion-compensated SE at systole, followed by in situ imaging of the same hearts in a contracted state. HA, TA, and sheetlet angle compared well between in vivo and ex vivo conditions[63] . A similarly high correlation between in vivo and ex vivo data was found in mice, using a diffusion-encoded SE sequence [9].
  Ex vivo studies of human hearts, together with those of other animals, have shown that myocardial microstructure is highly conserved across mammalian species. In inter-species comparison studies, ex vivo cDTI in fixed mouse, rabbit, and sheep hearts demonstrated similar transmural variation in HA [107]. A further ex vivo cDTI study of normal human, sheep, and rat hearts also found similar helical arrangements, as well as identifying a significant reorganization of cardiomyocytes in the remote zone of infarcted sheep hearts .[11] While hemodynamic and loading conditions cannot be precisely replicated ex vivo, these studies allow the heart to be imaged with far higher spatial resolution than can be achieved in vivo. For instance, ex vivo DTI has allowed the human atria and myocardial infarcts in swine to be imaged with submillimeter resolution [108], [109].
(ii) Independent verification of cardiomyocyte orientation and sheetlet orientation has also been obtained by comparing cDTI measurements with alternative techniques performed at various spatial scales. The techniques used for verification included histological measurements [24], [104], [110],[84] non-destructive MRI at the whole-heart level (e.g., high-resolution T2 weighted imaging [111] and 3D fast low angle shot imaging [112] using gadolinium-based contrast agents to provide contrast between myocytes sheetlets and cleavage planes) [113], and other non-CMR based technologies, such as synchrotron radiation imaging (SRI) [114], CLARITY with light sheet fluorescence imaging (LSFM) [115], and optical coherence tomography (OCT) [98]. For SRI, CLARITY-based LSFM, and OCT, whole-heart imaging was performed for small animal samples. It should be noted that ultrasound imaging can potentially be used to measure cardiomyocyte orientation with narrow windows of a transmural section [116].
(iii) Phantom studies are important for quality assurance and standardization in multi-center and longitudinal studies [68], [117], and can be used to support and validate methods development in MRI. The ideal phantom would be manufactured reproducibly, be stable for long periods of time, suited to repeat scanning, and evaluable using independent non-MRI methods. Isotropic phantoms comprising aqueous solutions of polyvinylpyrrolidone can be used to evaluate intra- and inter-center reproducibility in MD and FA [117]. More sophisticated biomimetic phantoms can be used to simulate the myocardial microstructure. One static phantom comprised discrete layers of co-electrospun hollow fibers simulating the orientations of cardiomyocytes and sheetlets [118]. The pore sizes were customized to match cardiomyocyte cross-sections and were validated using scanning electron microscopy. Dynamic phantoms include a compressible gel phantom [119], a rotating pig spinal cord [120], and an ex vivo lamb heart section under compression [121]. These can help clarify the sensitivity of cardiac diffusion signals to motion. However, their biological nature limits shelf-life and control over their microstructure, as well as exhibits fixation effects. Future phantom development promises to combine key elements of microstructure and motion, which will be invaluable to methods development and for facilitating larger multi-center studies.

Collectively, the large body of preclinical studies provides confidence that clinically derived cDTI indices, assessed with different pulse sequences and motion compensation schemes, are indeed reflective of the underlying microstructure of the heart. This is particularly true for the correlation of diffusion eigenvectors with cardiomyocyte/sheetlet orientation, where ground truth is easily defined. It should be noted that cDTI reflects the average microstructure in a voxel, but this has correlated very well with microscopic techniques that directly provide cellular resolution.

**Recommendations**

• A broad consensus exists that changes in microstructure in animal models are likely to closely mirror those seen in the human heart, and the use of preclinical models, particularly in vivo, to study myocardial microstructure is encouraged.
• While the consensus of this working group is that the accuracy of cDTI is well established, we, nevertheless, recommend that all preclinical studies exploit the availability of tissue for complementary analysis with one of the techniques described above.

### 3.12 Challenges and opportunities

Significant progress has been made over the last several years in pulse sequence design, analysis techniques, and first-in-man studies. Nevertheless, before cDTI can become a routine clinical tool, several challenges will need to be addressed. The biggest challenge at present lies in the inefficiency of this technique. When STEAM-based approaches are used, the TE of the sequence can be kept shorter than the T2 of the myocardium. However, the SNR loss inherent in a stimulated echo readout and in diffusion-encoding can result in noisy images. At present, most investigators perform around eight averages, requiring eight breath-holds, per slice. If images are acquired during diastole and systole, to examine sheetlet mobility, for instance, a total of 16 breath-holds may be required. While this can be easily justified in the research setting, greater efficiency will be needed for routine clinical adoption.

The SE-based cDTI techniques offer a mix of advantages and disadvantages. The TE required to accommodate the more complex gradient waveforms of these sequences is significantly longer than the T2 of the myocardium, resulting in a loss of signal and the potential for geometric distortion during the echoplanar readout. A similar number of averages, approximately eight per slice, is therefore usually required with both SE- and STEAM-based readouts. The principal advantages of the SE approach include its intrinsically higher SNR, reduced sensitivity to irregular cardiac rhythms, and capability to be performed during free-breathing. The image quality obtained with STEAM and SE-based techniques, when performed well, is similar and both provide extremely useful information in the research setting.

Several attempts have been made to improve the efficiency of cDTI. The blipped-controlled aliasing in parallel imaging approach has been used to excite two slices simultaneously [50], [51], [122], which allows a pair of slices (e.g., basal and apical) to be imaged per set of breath-holds. While technically appealing, the clinical/diagnostic value added with this approach has not yet been determined. AI-based reconstruction approaches to denoise cDTI, reduce the number of breath-holds required, and improve the accuracy of the estimated tensors are extremely promising [80], [123], [124]. With one such denoising approach, for instance, the number of breath-holds required could be reduced by 50% without compromising diagnostic accuracy [80]. Retrospective respiratory gating of free-breathing SE cDTI, using a low-rank tensor multitasking approach, has shown significant success [43]. Other retrospective approaches to gate free-breathing data, such as those based on principal component analysis and image entropy [51], [62], may also hold promise. The single-shot EPI readout used in both stimulated and SE cDTI is vulnerable to artifacts, and alternative readout schemes, such as spiral trajectories, are being investigated [54].[55] Reconstruction algorithms to reduce geometric distortion in SR cDTI images have also been described [81], [125], but more research will be needed in this area.

To the best of our knowledge, none of the commercial MRI companies currently have a prototype cDTI sequence available to their customer base. Likewise, none of the commercial postprocessing/analysis platforms have incorporated the ability to analyze cDTI data into their software. Despite the need for ongoing technical improvements, cDTI has reached a point where its broader dissemination is warranted. It is, therefore, the consensus of this panel that prototype sequences and analysis packages should be made available to the clinical and translational imaging communities.

**Recommendations**

• Both stimulated and SE-based sequences should be made available as works-in-progress packages, but the SE sequences should ideally be used on systems with gradients strength of at least 80 mT/m.
• Minimum duration breath-hold (low b and six diffusion-encoding directions) and longer breath-hold (low b and greater than six diffusion-encoding directions) versions of both sequences should be made available.
• Commercial vendors of a CMR analysis software should provide the ability to measure MD, FA, and the slope of the HA across the myocardium in their software.
• Commercial vendors are encouraged to collaborate with academic sites to facilitate the broader dissemination of platforms for retrospective respiratory gating of free-breathing SE cDTI datasets.

While greater sophistication is always possible, both during acquisition and analysis, we feel that the recommendations made herein will be feasible at most sites, facilitate standardized multi-center studies, and hopefully result in the addition of a large volume of high-quality data to the literature.

Wider dissemination of a simple and standardized acquisition/analysis approach for cDTI is a major goal of this working group. Simultaneously, we recognize that major needs and opportunities exist for ongoing technical innovation and development of cDTI, and place equal/major importance on this as well.

Much attention has, justifiably, been paid by the diffusion community to the maximal gradient strength on clinical MRI scanners, and commercial systems with maximal gradient strength of 200 mT/m are now available. These gradients should allow the TE in current M1M2-compensated SE sequences to be reduced significantly and/or more complex diffusion-encoding schemes to be explored. It should be noted, however, that with the b-values used in the heart, ramping the gradients up/down from their maximal value (Gmax) takes up much of gradient duration (d) and hence TE. In the heart, the slew rate of the gradients (200 mT/m/s) is, therefore, also a key factor. The slew rate limit has been required by the regulatory agencies, to prevent peripheral nerve stimulation and cardiac stimulation (arrhythmias). While recent work in swine suggests that far higher slew rates can be used without risk [126], the timeline toward clinical translation remains unclear. The availability of higher gradients and slew rates will facilitate the design of novel diffusion-encoding waveforms. Initial work in this area, using convex optimization, has shown promise [127], [128]. The use of higher gradients and novel diffusion-encoding schemes will require close attention to be paid to the effects of eddy currents [30], [128]. Initial work, however, suggests that the benefits accrued from a reduction in TE are not eliminated by the accompanying increase in eddy currents and, furthermore, that the effects of eddy currents in cDTI can be corrected for .[55]

We strongly encourage further work in the design of novel diffusion-encoding schemes, distortion correction, and eddy current correction. We simultaneously challenge the cardiac MRI physics community to develop multi-contrast sequences, analogous to fingerprinting, where T1, T2, and diffusion data could be simultaneously acquired [129]. Numerous opportunities for innovation exist in the postprocessing and analysis of cDTI data. New parameters, such as the recently described tractographic propagation angle [88], could provide new insights into myocardial microstructure. The integration of cDTI data with other measures of myocardial pathophysiology, such as electro-anatomical voltage maps [88], is also very promising. The use of cDTI to define a coordinate system (cardiomyocyte long axis, sheet, sheet normal) for 3D strain measurements of the myocardium is particularly appealing, and has the potential of allowing the fractional shortening of cardiomyocytes to be measured in vivo [130].

Diffusion MRI remains an extremely active area of research. While the techniques described above are very promising, the experience with most of them is limited and consensus recommendations for their use cannot be made at this time. The focus of this consensus document is on cDTI. It is worth noting, however, that other diffusion formalisms, such as IVIM [62],[58] and high angular resolution diffusion imaging techniques, such as diffusion spectrum and Q-space trajectory imaging [97], [131], have been used in the heart. Ongoing exploration of these formalisms is strongly encouraged.

In summary, patient-level cardiac DTI measurements have their basis in angstrom-level Brownian motion of water molecules. Influences between signal source and signal detected warrant further study outside the scope of this consensus statement to fully appreciate factors that may or may not be related to cardiac DTI-based inferences of the myocardium’s health or disease.

While much work remains to be done on enhancing hardware, pulse sequences, acquisition strategies, and postprocessing approaches, it is clear that cDTI is highly feasible, can be accurately and reproducibly performed and, most importantly, can provide unique insights into myocardial pathophysiology. We encourage the commercial vendors to provide the broad community with the tools and training to perform entry-level cDTI and encourage those with little experience with cDTI to engage more experienced sites, begin with simple acquisitions, and use this very powerful technique in a well-informed and responsible manner. We are confident that cDTI will soon join relaxometry-based metrics as a key tool in the CMR armamentarium.

## Funding

There are no source of funding for this work.

## Declaration of competing interests

Erica Dall’Armellina reports administrative support and article publishing charges were provided by the Society for Cardiovascular Magnetic Resonance. Andrew Scott reports a relationship with Siemens Healthcare that includes funding grants. David Lohr reports a relationship with Siemens Healthineers AG that includes funding grants. David E. Sosnovik reports a relationship with the National Institutes of Health that includes funding grants. Dan Ennis reports a relationship with Siemens Healthineers AG that includes funding grants. Dan Ennis reports a relationship with GE Healthcare that includes funding grants. Pierre Croisille reports a relationship with Siemens Healthineers AG that includes funding grants. Magalie Viallon reports a relationship with Siemens Healthineers AG that includes funding grants. Sonja Nielles-Vallespin reports a relationship with Siemens that includes funding grants and non-financial support. S.N.V. Co-author editorial capacity for JCMR—D.S. Associate editor co-author editorial board for JCMR—E.D.A. Corresponding author: editorial board for JCMR— The other authors declare that they have no known competing financial interests or personal relationships that could have appeared to influence the work reported in this paper.
